# Governing displaced migration in Europe: housing and the role of the “local”

**DOI:** 10.1186/s40878-020-00209-x

**Published:** 2021-01-25

**Authors:** Nasar Meer, Claudio Dimaio, Emma Hill, Maria Angeli, Klara Oberg, Henrik Emilsson

**Affiliations:** 1 School of Social and Political Science, University of Edinburgh, Edinburgh, UK; 2 Department of Social and Political Science, University of Calabria, Rende, Italy; 3 Mediterranean Institute for Gender Studies (MIGS), Nicosia, Cyprus; 4 Malmö Institute for Studies of Migration, Diversity and Welfare (MIM), University of Malmö, Malmö, Sweden

**Keywords:** Migration, Refugees, Local, Governance, Housing, Europe

## Abstract

This article will explore the extent to which a focus on the ‘local’ can tell us something meaningful about recent developments in the governance of displaced migrants and refugees. Taking a multi-sited approach spanning cases in the south and north of Europe, we consider how the challenge of housing and accommodation in particular, a core sector of migrant reception and integration, can shed light on the ways local and city level approaches may negotiate, and sometimes diverge from, national level policy and rhetoric. While it can be said that despite variation, local authorities are by definition ultimately ‘always subordinate’ (Emilsson, Comparative Migration Studies, 3: 1-17, 2015: 4), they can also show evidence of ‘decoupling’ across geographies of policy delivery (Pope and Meyer, European Journal of Cultural and Political Sociology, 3: 280–305, 2016: 290). This article traces how possible local variations in different European cases are patterned by ground-level politics, local strategic networks, and pre-existing economic resources in a manner that is empirically detailed through the study of housing.

## Introduction

According to the International Organisation for Migration (IOM), over 1.6 million displaced migrants and refugees entered Europe in 2015 at the height of the so called refugee crisis (IOM 2015). By the middle of 2020, COVID-19 largely brought the movement of people to a halt as individual states and whole regional blocs introduced travel restrictions. Some have been more cautious than others in accounting for international laws and conventions intended to protect the right to entry for refugees and asylum seekers (Meer and Villegas 2020), but the general pattern is one in which the pandemic has made an unfavourable climate for displaced migrants and refugees worse (Meer et al. 2020).

While reason might dictate that the reference to a ‘crisis’ should describe the circumstances of ‘those fleeing devastation, or to those trapped in it’ (Bhambra 2017, p. 395), the ‘refugee crisis’ came to refer to how European receiving states have struggled with the social and political implications of this movement of people. At one time characterised as the ‘new normal’ by the European Commission (2016), and despite the principle of sharing numbers of people according to relative economic strength and country size, as outlined in the EU Task Force for the Mediterranean and wider EU Strategic Guidelines, particular localities have been forced by necessity to innovate in managing the arrival, flow and accomodation of people.

In this article we detail and discuss some of the emergent modes of governance that developed during this period and which can be observed in our study of southern and northern European localities. We are especially interested in two tendencies. The first is that local and city level articulations of migrant and refugee reception negotiate national level policy and rhetoric. This contributes to a burgeoning literature ‘emphasising mediation and local-level negotiation … in the development of immigrant policies in European cities’ (Borkert and Caponio 2010, p. 19; cf. de Graauw and Vermeulen 2016). In our case illustrating features of ‘decoupling’ across geographies of policy delivery (Pope and Meyer 2016, p. 290), this local variation is patterned by ground-level politics, local strategic incentives, and pre-existing economic resources in a manner that requires further investigation through live cases. The second is that local and city level forms of reception of displaced migrants and refugees are the sites of early ‘integration’, but that this especially relies on associations from the third sector which have assumed a key role in what Elia (2013, p. 36) has termed ‘bottom up welfare’.

How the key area of housing provision features in these two dynamics is important, espeically where the national state is relying on third sector partnerships. The paper therefore discusses local responses to displaced migration as an issue of *governance* which entails dispersed networks based on partnerships, and the blurring of boundaries between state and civil society (Elia and Fantozzi 2013). With ‘less emphasis on hierarchy and the state, and more on networks and markets’ (Bevir 2011, p. 1), this is different to seeing the arrival of displaced migrants and refugees as purely a matter of central *government*. It is an established distinction that is crucial to grasp where the governance of migration relies on a ‘mixed economy’ of welfare provision that involves working with voluntary and community sector organisations, and NGOs at various levels through service delivery, consultation and partnerships, and which are especially evident in the reception and integration of new arrivals (McDaniel 2016). This broad tendency is accentuated in austere economic times, where the diffusion of state responsibility to civil society may also be understood as a means of softening the impact of reduced public spending.

Taking a multi-sited approach spanning the south and north of Europe, the article considers the ways in which the governance of housing and accommodation, a core sector of migrant reception and integration, proceeds in local municipalities. In so doing, the article seeks to add to (rather than reject) prevailing approaches that offer ‘national model’ explanations, specifically by discussing fieldwork in medium sized urban areas. This includes the conurbations of Cosenza (Italy), Glasgow (Scotland), Malmö (Sweden) and Nicosia (Cyprus) that have in common experiences of the recent arrival and settlement of displaced migrants and refugees. In what follows the article begins with a discussion of migration governance and housing, establishing the crucial relevance of this topic before turning to a discussion of case selection and the methodological approach. The article then moves to outline to key findings. The first is to show how local approaches are characterised by sometimes contradictory impulses of dispersal rather than devolution per se, the second is to highlight the increasingly central feature of non-state actors in this process. The article concludes by discussing some of key implications for the governance of migration and accomodation more broadly.

## Migration governance and housing

In her discussion of contemporary citizenship regimes, Bloemraad (2017, p. 29) makes the observation that localities may represent ‘a new frontier for migration studies’, a hypothesis that seems ripe for study in light of the frontline response of cities and regions to the subsequent movement of refugees across the Mediterranean. For example, and reflecting the greater role of horizontal and not just vertical governance relations, research focusing on the response of localities in Germany reported that:

Even though cities are tied into the implementation of federal politics of migration control (through registry offices, social services departments, schools, etc.), and even though in most countries they do not have legal competence to care for asylum seekers and refugees, the recent scale of arrivals and the slow reaction of national authorities have often left cities at the forefront, forcing them to play a role without having either a legal mandate or any specific budget to do so (Mayer 2018, p. 234).

Recognising this tendency, and the prevailing role of contingency, in their guidance to the Council of Europe (CoE), the Congress of Local Regional Authorities (2017, p. 1) maintained that while ‘local and regional authorities are the cornerstone of efforts to effectively tackle the current refugee situation’, there is a prevailing disconnect between objectives of national governments and practice in local authorities. ‘What is sorely needed’, they argued, ‘is for national governments to provide local and regional authorities with the legal tools, the necessary legal framework and the infrastructure they need in order to develop their action’ (ibid., p. 14).

There is a degree to which this might be understood as part of a longer development in what Brenner (2004) has previously characterized as the ‘rescaling of statehood’. In this view, there is already an existing trajectory that might equally be the basis of a normative approach. For example, and in different ways, Jørgensen (2012) and Scholten (2013) have previously pointed to a ‘decoupling’ between the local and national levels, observing a tendency elsewhere characterised by Myrberg (2017, p. 324) as ‘local governments … shifting from a passive to an active role, not only in the sense of implementing policies, but also as sources of innovation and of new frameworks of relationship with other levels of government’. This is not incommensurate with national level governance, but it does stress that cities and local authorities are not merely the sites for national-level processes to filter down (Nicholls and Uitermark 2016), but on the contrary have a distinctive role to play in the design and implementation of refugee and integration policies (Filomino 2017).

Of course it is easy to overstate these dynamics, and we would wish to guard against a simplistic assumption that the local can address the limitations of the national. Indeed, our findings suggest that local level room for manoeuvre may well be limited and very partial in the area of accommodation provision and support. No less significant, the evacuation of the national state from local provision can leave the latter incapable of ensuring even elementary provision. At its strongest, we might heed Emilsson’s (2015, p. 4) insistence that local authorities are by definition ultimately ‘always subordinate’, and no less appropriate was Bauman’s (2003, p. 37) insistence that ‘there are no city-centred, let alone city-confined solutions to systemic contradictions and malfunctions’. A cautionary reminder against some of the more panglossian statements about cities presented in places such as Barber’s (2014) influential thesis *If Mayers Rules the World*, Bauman (ibid., p. 38) reminds us, ‘the city is as well the training ground where the means to placate and disperse that uncertainty and insecurity can be experimented with, tried out and eventually learned and adopted’ (ibid., p. 37). In this article, we offer an analysis of the relationship between migration governance and the local in respect of housing and accommodation in our cases.

As detailed in the next sections, refugee accommodation and settlement processes are a contextually rich means of gauging forms of vertical and horizontal partnership between stakeholders amongst our cases. While there is a burgeoning literature addressing the housing and accommodation trajectories of displaced migration and refugees (e.g. Flatau et al. 2015; Francis and Hiebert 2014), it is often material that is single case focused and ethnographically driven – both of which are assets in understanding the migration experience. Less well established however are multi-case policy governance accounts, especially charting contemporary developments (cf Peace and Meer 2019). This is surprising since the academic and policy literature share in common a view that ‘housing is a fundamental question to be faced from both short-term and long-term perspectives’ (CLRA 2017, p. 33).

What is short term and what is long term are not questions that stand apart from the wider migration experience of formal and informal legal status, and the variously conceived ambitions for integration therein. As a key sector of integration in Ager and Strang’s (2008) well known formulation of integration, housing is in many respects the cornerstone of the needs of displaced migrants and refugees. Of course any discussion of a ‘home’ as something greater than ‘housing’ tips into a rich literature which, following Brah (1996), recognises the idea of a home as a ‘lived experience of a self in a locality’. In a related way, Fadlalla (2011) discusses how refugees themselves conceptualise the meaning of home, Kissoon (2015) explores the idea of home as ‘intersections of displacement’, and Smith's  (2015) focus on refugee material culture and homebuilding is also very necessary here. While the focus of this article is more the technical features of policy provision rather than the affective sense of home, each of these framings have a bearing. As Phillips (2006, p. 539) put it some years ago:

The housing conditions and experiences of refugees clearly play an important role in shaping their sense of security and belonging, and have a bearing on their access to healthcare, education and employment. The ability to access safe, secure and affordable housing is also likely to have an impact on community relations, the level of secondary migration by refugees, and the development of a migrant household's capacity for secure and independent living.

As we show below, what needs to be added to this is that accommodation is often used as a deterrent for migration rather than as a facilitator of incorporation or integration. The location and quality of accommodation, the triaged nature of its provision or refusal, and the temporary tenure of contracts and forced evictions speak to this tendency. All of these can be a feature of an asylum seeking journey, something that often begins with a process designed to hold new arrivals in locations distant to eventual accommodation.

## Methods and case selection

While our four urban areas are broadly comparable in terms of their size, their experiences with migrants and refugees differ considerably. Each case is what Glick Schiller and Caglar (2009) may term low-scale cities, for they assume a weak position in global exchanges and are – or were - dependent on particular industries and sectors. Yet despite being non-global cities, they are relevant because while Nicosia and Cosenza are landing points for many refugees as they first enter the EU, Glasgow and Malmo are  more like final destinations. Displaced migrants have been accommodated in Glasgow since the mid twentieth century; however, in the main this was achieved through managed refugee resettlement programmes, which brought small numbers of recognised refugee groups to Scotland (Piacentini  2012). This changed significantly with the Labour Government’s UK the Dispersal Scheme, which was intended to 'share' accommodating asylum seekers across the UK (Hynes 2011). To-date, Glasgow is the only local authority in Scotland to have participated in the Dispersal Scheme, and is estimated to accommodate up to 2000 asylum seekers annually, the largest intake for a single local authority in the UK (McAllister 2015, p. 244). Malmö and Eslöv, meanwhile, are two municipalities in the Scania region as well as part of the Öresund region that connects Sweden to Denmark and the European continent. The distance between the two municipalities is close, only 20 min by train but there are key differences: Malmö is the third largest city in Sweden, located on the border to Denmark whereas Eslöv has a small city center as well as a rural conurbation. In contrast, as indicated, Calabria has become a place of almost uninterrupted arrival, with the number amounting to about 5 % of the regional population. This follows long periods of emigration to the USA, Brazil, Canada and Argentina and to north-eastern Italian regions (Fantozzi 2011). Finally, in Nicosia, new arrivals and applications for asylum have created novel challenges because of the absence of sufficient legislative and administrative structures. According to some reports, the majority of asylum seekers enter Cyprus via the occupied areas in Northern Cyprus, crossing the ‘Green Line’ (marking the separation between the Republic of Cyprus and Turkish controlled Cyprus) to submit an asylum application (Cyprus Refugee Council 2019). Although there are authorised points of crossing along the Green Line, these are not considered official entry points into the Republic of Cyprus (RoC).

Our discussion of these cases relies on a qualitative approach to data collection and analysis, and recognises that researchers are working in ethically fraught and logistically complex arenas. The inquiry therefore reports on specific character of accommodation and integration approaches, rather than purely uncovering the frequency with which those conditions and their outcomes arise. This relies on eighty-eight interviews with stakeholders including local, national and non-state actors, conducted via face-to-face meetings and audio-recorded as far as possible. The country and sectorial breakdown is detailed in Table 1 below.

**Table 1:** Interviewees according to sector and case

Level/sector	Malmo	Calabria	Nicosia	Glasgow	***Total***
The national level policy actors	2	3	2	3	*10*
The regional level policy actors	2	3	1	6	*12*
Professionals working with refugee reception at the local levels	10	6	3	16	*35*
Asylum Seekers and Refugees	20	4	1	6	*31*
					***88***

Interviews were transcribed or documented using agreed formats and standards for handling the issue of multiple voices, interruptions, labelling of participatory and visual activities, and so forth. The research did not set out to test theories in a narrow sense but instead explore theories and concepts about migrant and refugee integration from empirical data. It used an inductive and iterative approach that had the necessary openness for finding new and surprising practices. Field notes and transcripts were organised using NVivo qualitative data analysis software, to enable us to connect relevant themes and patterns in the data. While field notes were provisional and not meant to be systematic they helped the coding to be developed inductively, using themes that emerge from the data rather than being imposed in advance. In some places, especially the discussion of Nicosia, there is greater space for a richer description of the field sites precisely there is less governmental literature upon which to draw up. In what follows, the discussion sets out some of the prevailing conceptual means through which the local and national governance dynamics can be understood as operating with respect to displaced migration. After this, the specific characteristics of the empirical cases under study are drawn, with a particular emphasis on general features and tendencies that are notable across localities. Subsequently, we seek to highlight two prevailing features that span our respective cases, and which focus on specific issues related to housing, but in ways that distil our broader concerns with migration governance. The first concerns the dynamic of local and national governance, and specially where accommodation policy is being pursued within the parameters of existing governance arrangements. The second relates to the role of non-state actors, be they voluntary, charity or private providers, for the objectives of migration housing policy.

## Localism: devolving or dispersing?

Across our cases, a first observation is to distinguish between the formal arrangement of national and local governance, before we can assess how this contains or is exceeded in the practice of housing provision. Building on an existing decentralized reception network, involving municipalities and third-sector organizations that have been in place since 1999, at the national level the Italian SPRAR system came into being in 2002 as an Italian Ministry of the Interior funded collaboration with National Association of Italian Municipalities (ANCI). In the SPRAR model, local authorities which choose to participate in the SPRAR network apply for short term (three-year) grants for projects to pursue ‘integrated reception’. This aspires to span education, employment, and cultural activities (Camera dei Deputati 2018). As one national level respondent it Italy outlines:

Since the National Asylum Plan, this system has been a bottom-up model. Some local administrations, which had already considered collaborating with the third sector associations, have promoted through ANCI the idea of a common front in the reception management. After a short phase of experimentation, the idea of a network, based in the provinces and municipalities, came forward [...] with a central office that would create common standards and maintain continuous dialogue with the individual territories across all projects.

This is the way in which the so-called integrated reception was conceived: facilitated by the national level Central Service, as a technical structure of the Ministry of the Interior, and allied to policy development in the territories. The multilevel dynamic is a key feature of what became known as ‘second reception’, as described by a local level another stakeholder as ‘working in collaboration with local and regional associations … to decide where to allocate the welcomed migrant according to their specific case and availability of places in territorial projects’.

According to the SPRAR Data Bank (2018), there are 35,881 people in the SPRAR system, of whom about 3500 thousand are unaccompanied minors. There are 877 active projects in Italy involving 754 local authorities, most of them municipalities. The number of migrants hosted within the SPRAR system is higher in the South than in the North and Center of Italy, and in the Locride area, a rural zone inland from the Jonic coast (where the reception of refugees has become an important part of local development). In those municipalities, the displaced migrant population is above 15% (ISTAT 2015). The Municipality of Cosenza has managed a SPRAR project since 2009. The main association responsible for implementing the services is La Kasbah cooperative, which provides services for asylum seekers and those recognised as requiring humanitarian protection, single men and families.

Recent political changes, however, have thrown the SPRAR model into uncertainty, and especially its capacity to move beyond the temporary ‘extraordinary reception centres’ (CAS). Until the 2019 ‘Salvini decree’, asylum seekers passed through reception centres and were assigned to a SPRAR project. Now, asylum seekers remain in a state of first reception, because only those in receipt of refugee status may leave CAS centres. The old SPRAR system has been renamed SIPROIMI (Protection System for beneficiaries of international protection and for unaccompanied foreign minors) and is aimed only at refugees who have already received a positive response to the asylum application (refugee status or subsidiary protection), or unaccompanied foreign minors. It is too early to anticipate the consequences of this, but it is widely recognised by local stakeholders that the reception system solution is inadequate compared with the accumulated good practice of the SPRAR approach (Algostino 2018).

The bottom up approach of Calabria is most obviously contrasted with more top down approaches in Malmö, though the latter has local imperatives too. The national level 2016 Settlement Law, for example, makes it mandatory in Sweden for all municipalities to receive refugees including organising their housing. In this fashion, the numbers of refugees received by the county and municipality is suggested nationally by the Swedish Migration Agency, but the decision on the form of reception is made by each county, and is related to the size of the population, prior experience of refugee reception and estimation of wok opportunities. It is important to note that most migrants settle themselves, without any state or local control, and that a large majority of the municipalities accepted organised settlement long before the Settlement Law. The main impact of the national level law has been a greater number of refugees in the municipalities with historically lower numbers of refugees. As civil servants at the national level Migration Board acknowledge:

Before 2016 the Employment Service made agreements with the municipalities. So they [municipalities] said “we can receive five” or “we can receive three” and so on. The number was never enough, so we never got them settled. There were people who remained in our [the Migration Board] systems for years and not out in the community.

Prior to this, the national government and local municipalities would negotiate the numbers of arrivals. The incentive for local government was economic, because they received funding for every settled refugee. More vulnerable refugees were deemed harder to settle due to the complexity of need. The central–local relationship after the 2016 law, however, is characterised more by directives than by negotiations. As another civil servant puts it: ‘Now there is nothing to negotiate. We cannot negotiate a law’.

The centralised nature of the law can be a misleading indicator of its impact, for it has been received very differently in each municipality, something reflected in housing standards, costs and the length and form of accommodation contract. So while it is true that ‘local integration policies must acknowledge that cities do not operate in a vacuum’ (de Graauw and Vermeulen, 2016, p. 990), and that ‘[n] ational policies have an impact on city policies’ (ibid), in this case the centralisation of power over settlement has resulted in a higher capacity but also greater differentiation in housing arrangements on the local level. Stakeholders in the County Administrative Board that coordinates and monitors accommodation settlement, acknowledge that many local housing solutions are short term, for ‘[m] ost [municipalities] have no sustainable and long-term solutions, perhaps just under half of them’ (interview). Indeed, municipalities tend to offer two or, like Malmö, four year temporary contracts, while others only provide solutions for one to 6 months. The key implication of the law is that it has required a shift of responsibility from a national to a local level, leaving concrete decisions and issues of equality of integration for local agencies to decide. It has moreover made visible a competition for resources. One of the consequences is that the needs of different categories of homelessness are set against each other, in some municipalities more than in others, in places where there is political contestation over housing of refugees.

This relatively straightforward landscape in Malmö becomes immensely more complicated when we shift our focus to Glasgow. Here responsibility for both the UK level Dispersal Scheme (which oversees the accommodation of asylum seekers) and the VPRS (which oversees the resettlement of Syrian refugees) is ultimately held by the Westminster Government through the Home Office. However, the schemes rely on noticeably divergent formalised arrangements between UK, devolved and local government for their implementation. The provision of Dispersal accommodation, once managed through a direct relationship between the Home Office and participating local authorities (in this case, Glasgow City Council) is also part of a profit seeking non-state actor partnership. Since 2012, contracts for accommodating Dispersed asylum seekers is have been awarded to private companies, which have subcontracted local private and Registered Social Landlords (RSLs) to provide and manage asylum seeker housing.^1^ The privatisation of Dispersal contracts has not only removed the day-to-day provision and administration of housing for asylum seekers from local authorities, but also policy competences – i.e. the type and location of Dispersal housing, sizes of asylum seeking populations within locales, and housing standards – which would otherwise come under local urban planning remits. As a result, Glasgow City Council stakeholders observed, current iterations of Dispersal involve little policy consultation with local authorities, who feel removed from centralised decision-making processes. ‘I don’t know why they asked us for our input’, stated one stakeholder, ‘as if they were listening, because they weren’t’ (interview).

Though housing issues are devolved to the Scottish Government, matters relating to immigration are reserved to the UK Government. Here the multi-level settlement comes into tension, for because asylum is considered primarily an immigration matter the UK Government does not involve the Scottish Government in Dispersal housing issues. However, a notable exception to this precedent is the issue of asylum seeker homelessness and destitution, which can occur if an asylum application is rejected and a person is evicted from their accommodation in Glasgow. Recent enquiries have started to consider the extent to which destitute asylum seekers may fall under devolved policies; however, approaches are being made with caution. A devolved government stakeholder illustrates the dynamic:

And so we don’t get involved in their [Home Office] accommodation issues. But obviously if somebody’s gone through the system and hasn’t got a positive decision then the destitution sort of aspect of it will be [ … ] within the remit of Scottish Government but it throws up issues [ … ] But the position of Scottish Government can’t be that we fund directly provision for people with no recourse to public funds. It’s a reserved matter therefore we’ve got no locus or remit to actually act on it.

In Glasgow then, gaps in accommodation provision created by the formalised Dispersal governance infrastructure – such as the issue of asylum seeker destitution – have been highlighted and filled by robust third sector and grassroots networks. The more recently implemented VPRS has offered a slightly different governance dynamic to Dispersal. The Scheme is overseen and funded by the Home Office, but is run in agreement with participating local authorities, which have decision-making competences over housing type, location and suitability. This has enabled local authorities to mobilise their local expertise in housing provision and make it responsive to the needs of both refugees and the local area. In Scotland, this has resulted in a fairly localised approach to housing provision. For instance, where Glasgow City Council mobilises its existing homelessness infrastructure to house VPRS refugees, Aberdeenshire Council has utilised excess private accommodation, which has become available following a local economic downturn. Whilst the Scottish Government has ostensibly been more involved in responding to Syrian Resettlement, like Dispersal, the VPRS is primarily an agreement between local actors and central government.

In Nicosia, meanwhile, we find a further contrast of local-national enunciations, for when it comes to reception and integration policies, at the National level Cyprus seems to be the maintaining centralised asylum services. At the state level, the present national level Integration Task Force (UNHCR 2019) aims to ‘contribute to the development of a national integration strategy’. Its members include refugee associations, local authorities, and business community representatives. The initiative seeks to encourage 'open dialogue' with all actors involved and could prove crucial in the future development of a national strategy and action plan. At the moment then, national level integration policies in Cyprus are still centrally controlled by the government, and it is a centralised system that has engendered gaps in addressing the needs of asylum seekers and refugees. This is the point where local authorities and NGOs step in to play a crucial role in their integration. As one NGO representative describes:

The social welfare office that is supposed to be responsible for housing cannot meet the needs of asylum seekers. If someone calls the office and tells them, “I have nowhere to stay”, they cannot handle the case. Therefore, NGOs step in to find a solution.

Historically, Cyprus has had a relatively low number of asylum seekers due to its geographical isolation from mainland Europe, and its exclusion from the Schengen area. Consequently, the first asylum application was only registered in the late 1990s (Spaneas et al. 2018). The shift really occurred in the 2000s and more recently as other routes into Europe have become less accessible. As such between 2016 and 2018, most asylum seekers in Cyprus originated from the Syrian-Arab Republic, India, Pakistan, Bangladesh, Vietnam, and Egypt.

The prevailing place of national level accommodation is offered in the Kofinou Reception Centre which is located in an isolated, rural area and is the only one on the island. At an earlier stage, the Asylum Service also established a temporary reception centre in the Kokkinotrimithia region with the help of EU funding. While Kofinou is meant for adults, a number of families with young children are also housed there, and are theoretically allowed to stay for a maximum of 6 months.^2^ However, they are often forced to stay longer as private housing is difficult to secure. There are currently 5263 asylum seekers in Cyprus but the centre’s capacity is only 350 people, meaning that other than the 265 people hosted in Kofinou and the 130 unaccompanied children residing in special shelters, the vast majority of applicants live outside the centre. However, there are no statistics as to where and under what conditions asylum seekers live and other crucial data.

The centre is grouped into one area for families, and one gender-segregated area for individuals. The two family rooms have four beds each connected by a small hall and a private bathroom, while the individual’s area has two communal washrooms. The close proximity of these amenities has failed to consider gender based vulnerabilities, including the possibility of sexual harassment or assault. There are eight common spaces: four cooking units, a study room, a library, a children’s activity room, a clinic, and a playground.^3^ The centre also provides two daily meals, breakfast supplies, free bus transportation, a social worker, a psychologist, and an interpreter (Spaneas et al. 2018). Residents of the centre have a card for free hospital care, while the children can attend school nearby. However, asylum seekers’ allowance is usually 2 to 3 months late and is insufficient to cover basics such as toiletries and school supplies (Spaneas et al. 2018). Recreational activities are organised sporadically by NGOs as the government doesn’t provide any funding outside the framework of their approved projects (UNHCR, Cyprus 2018).

### State actors or non-state actors

A second feature that spans our respective cases, and which focuses on specific issues related to housing but in ways that distil our broader concerns with migration governance is the role of non-state actors. Amongst others, Ferguson and Gupta (2002, p. 989) discuss the ‘transfer of the operations of government’ toward ‘non-state entities’ in ways that are especially true of market dependent provision such as housing. One implication, as Blanco et al (2014, p. 3133) discuss, is how the role of non-state actors means that states can govern by ‘simultaneously retreating and advancing’ in so far as ‘withdrawal from direct service delivery [becomes] matched by its advance into regulation of service delivery by others’.

What has previously been empirically charted by others, including Esteves (2008), Campomori (2005) and Morén-Alegret (2002) focuses more on how NGOs, charities and third sectors organisations stand outside the state to provide ‘various services and offer political support for immigrants’ rights claims’ (Borkert and Caponio 2010, p. 19). This is a feature of our cases too but what is especially relevant is how these non-state providers function as almost actors in both the design, funding and implementation of their activities.

In Calabria, for example, third-sector organisations play a leading role in the identification of suitable accommodation, whether it comprises individual apartments or group accommodation, both small (about 15 people), medium (up to 30 people) and large (more than 30 people). This is especially the case in identifying “independent” accommodation solutions which, in Italy, are mainly managed by the private housing sector. However, the SPRAR project also tries to be involved in this, with the promotion, support and possible mediation between the beneficiary and the owner, during the negotiations on the rental contract (e.g., agreements with real estate agents, assistance in relations with the owners). Indeed, private apartments are mainly used, which represent 90 % of the available accommodations. In the apartments, refugees, asylum seekers and beneficiaries of subsidiary protection could stay for six months (extendable for a further 6 months) during which time they were supported by social workers to find an independent accommodation. A SPRAR operator explained:

I became a housing insertion assistant after being a beneficiary. Many times, I’m the first person to interact with the migrants who arrive in the SPRAR project. [...] We help to look for an accommodation even after the reception period but also we mediate with some small problems of cohabitation that may emerge within the community.

The direct transition from housing managed by the reception centre to independent accommodation can be difficult. Intermediate or temporary housing solutions are often used as a stepping stone (shared apartments with other guests, rented rooms, collective social housing). Unsurprisingly this varies according to needs and circumstance, as one stakeholder describes:

Generally, we have more problems in finding accommodation for families. It's easier for individuals. Our role is also to reassure people who rent an apartment about the regularity of payments and migrant assistance.

In Sweden, there has traditionally been greater resistance from civil society organisations to undertake what they see as the responsibility of the public sector. Nonetheless, a greater degree of participation from non-state groups in Swedish civil society is notable, specifically in the joint initiatives between municipal and civil society actors associated with the Refugees Welcome Housing project. This took inspiration from the German project *Mensch Mensch Mensch*, which matches refugees with landlords via a website and local meetings. The city of Malmö deemed this an example of best practise and started to collaborate more closely with the non-state partners involved, as described by the following respondent:

The city of Malmö realized that their housing solutions was insufficient. That they did not have enough, like in many other municipalities. And that they were looking for different possible solutions. And they thought our concept was interesting and contacted us. So we were approached by their manager in the labour market and social administration and asked if we were interested to work together.

Together, the city of Malmö, with a smaller municipality in the countryside and Refugees Welcome Housing applied for extra funding, which they successfully received. There is however a particular focus to this collaboration. This is that it tends to focus on those displaced migrants and refugees which the municipality has no formal responsibility for, such as families in need of more suitable housing, or asylum seekers and undocumented migrants. In many respects, this is an example of the multi-partner local network which pools skills to cooperate on cases that exceed the single capacity of one stakeholder. So even if there is formal cooperation with the city of Malmö, their activities are similar to other civil society organisations, who tend to prioritize the most vulnerable migrants that fall outside the formal responsibility of the public sector. From the perspective of the civil society there are, therefore, tensions in relation to what is acknowledged as part of a strong welfare state and a resistance to replace that responsibility with actions from the civil society (Osanami Törngren et al. 2018). Indeed, non-state actors more commonly assume a role through the option of residence in ‘own accommodation” where the person can choose a municipality to reside. The Swedish government had estimated that ten percent of asylum seekers would choose ‘own accommodation’ but in reality the figure is closer to 60 %. Many asylum seekers moved to the cities where family and friends resided, often to areas that are economically poorer. In 2018 the Swedish Government proposed that the own accommodation option would be reduced only to those who have the financial means and can prove access to housing (Employment Ministry 2018).

In Glasgow, the privatisation of Dispersal accommodation contracts has reshaped both vertical and horizontal governance dynamics. The model of governance under which the contracts operate has been described as ‘decentering’ (Darling 2016), a system in which central government retains decision-making power, whilst (1) outsourcing operational and political responsibility to local stakeholders without (2) providing a system through which local stakeholders can input into existing systems. As we note above, this model has led to frustration amongst both local government and third sector stakeholders, who feel that they have been left to resolve system issues without support, and in the knowledge that their solutions can be undercut by central government.

At the same time, the privatisation of Dispersal contracts resulted in a high degree of complexity to housing processes, involving multiple stakeholders from different sectors. This complexity has necessitated the development of relationships between private, public and third sectors, especially at points of transition. For instance, if an asylum application is successful, the new refugee (accommodated for now in private Dispersal accommodation) will be obliged to seek accommodation from local government. However, the transition between privatised Dispersal housing and social housing has been fraught, and has left new refugees vulnerable to homelessness, and placed both Glasgow City Council and third sector organisations under pressure. Other organisations have developed their own internal initiatives to combat the problems and gaps in provision left by Dispersal governance – for instance, Positive Action in Housing’s ‘Room for Refugees’ Scheme, a scheme similar to Malmö’s Refugees Welcome Housing Project, which asked volunteers to provide a spare room for asylum seekers facing homelessness.

This is in marked contrast to Cyprus where, local authorities lack a dedicated budget to implement integration activities in their daily work and are therefore restricted to working within the parameters provided by the national government. To get around this restriction, they participate in EU or national projects, implementing initiatives like language courses and other integration initiatives. As a municipality representative explains:

Municipalities sooner or later realised that they needed to find solutions on a local level and get over the legal restriction regarding access to funding. Therefore, they have begun to establish collaborations with NGOs or even started affiliated NGOs in order to access EU or Nordic funding to implement integration programmes … Unfortunately, as the available funding is on a project basis we always feel that the plug can be pulled at any moment!

As stated above, the funding is project based, therefore integration projects are often short-lived and unsustainable. Another municipality representative said:

The municipalities are designing projects from childcare services to language courses, but these are all projects with an expiration date, while the needs of refugees are ongoing.

NGOs are also paramount to the integration process, but funding remains a huge obstacle. The state essentially buys services from the third sector to implement key activities rather than encourage decentralised actions. The Local Council of Volunteerism of Kofinou (SKE) and social workers are in charge of the daily management. The SKE staff are not routinely trained on asylum-related issues or gender issues, and are not provided with psychological or other support, ultimately adversely affecting both the staff and the asylum seekers. Arabic-speaking interpreters visit the centre daily and a French interpreter makes weekly visits, but according to the UNHCR, this is insufficient. MiHub is a programme co-funded by the European Commission from the Asylum, Migration and Integration Fund (AMIF) (90%) and the Republic of Cyprus (10%) which aims to provide practical assistance, support and information to migrants. The project employs sixteen social counsellors in help centres across the four major cities in Cyprus, who have provided guidance to over 3000 migrants on issues such as employment, housing, education, and social benefits.

## Conclusions

Taking a multi-sited approach spanning the south and north of Europe, this article has considered the ways in which the governance of housing and accommodation, a core sector of migrant reception and integration, proceeds in local municipalities. It is a contribution which sits amongst previous research into the governance of displaced migration that emphasises *either* bottom-up participation of grassroots citizens or top-down influence from the state. While important, both of these perspectives are limited because civil society is composed of mediating institutions that blur distinctions between top and bottom (O’Toole et al. 2016), such that it is crucial to bring together top-down and bottom-up perspectives together into the study of migrant and refugee reception (Cornwall and Coelho 2006; Lowndes and Thorp 2010). What this discussion has tried to do is to empirically add to our understanding of such governance dynamics, specifically by elevating an account of ‘local’ in terms of civil society and stakeholder engagement in the accommodation of displaced migration. In so doing the article seeks to add to (rather than reject) prevailing approaches that offer ‘national model’ explanations, by discussing fieldwork in medium sized urban areas. As de Graauw and Vermeulen (2016, p. 990) remind us, it is cities that ‘administer more of the programmes and deliver more of the services critical to immigrants’ daily lives’. This article has charted this in the conurbations of Cosenza (Italy), Glasgow (Scotland), Malmö (Sweden) and Nicosia (Cyprus) that have in common experiences of the recent arrival and settlement of displaced migrants and refugees. In so doing the discussion has tried to populate with live cases a concern that can become somewhat de-ontological when pursued principally theoretically.

To this end what the study finds is that successful approaches need to span both mode one (emergency reception) and mode two (long-term settlement) accommodation. While national level policies are principally interested in the former, it is the more local level initiatives that are able to deliver the latter. The local is therefore a space in which the relationship between civil society and governance becomes a vantage point to observe forms of social (and other) capital at work in migrant and refugee incorporation. In this scenario, and ‘whatever the national framework of immigrant incorporation policies’ (Ambrosini 2017, p. 597), the urban level needs to be appreciated as a policy-making field in itself’. Where national and supranational responses appear bound by political intransigence,^4^ the role of civil society is prominent in emerging research about how local actors are accommodating recent arrivals (Lacroix and Desille 2018). As the SPRAR system illustrates, however, if ‘accommodation’ is indeed to become a ‘home’, then this requires a joined up approach with labour market participation and other spheres (including the civic sphere through language support) too. This should not imply that the answer rests only in greater devolution. As the Nicosia case illustrates, when the national state vacates the space, there is no guarantee that the local state can fill the gap. Moreover, and as the Glasgow case shows, at present the two tier system which corresponds to asylum seeker and refugee status respectively, undermines the whole system because of the social problems it exacerbates rather than addresses. Despite sharing similar experiences and exhibiting similar needs, those on the asylum and refugee track can enjoy radically different social welfare services. It is principally in these circumstance that voluntary stakeholders become central to current provision across the cases. Much of the effort of management weighs on the capacity of third sector associations and NGOs to create networks with private actors, with the support of the public administration. In this respect, governance creates partnerships, networks and groupings that give participants a new basis for defining who they are and what they are trying to achieve. As Ambrosini (2017, p. 594) elaborates,

What is important is that civil society organisations do not confine themselves to easing tensions between state sovereignty and the affirmation of universal human rights: the controversial issue of protecting irregular immigrants has in some cases given rise to forms of protest, advocacy movements, or mobilisations by undocumented residents themselves.

Not all of these challenges are about migration. The unsatisfactory housing situation among asylum seekers and refugees mirrors a more general situation with a shortage of affordable housing across our sites. Addressing the former, therefore, to some extent involves addressing the latter, a challenge of considerable scale not to be misattributed.

## Data Availability

All the data is available at www.glimer.eu

## Abbreviations

GLIMER: Governance of Local Integration of Migrants and Europe’s Refugees

## Footnotes

1 Between 2012 and 2018, accommodation was contracted to multinational, Serco. As of 2019, the Mears Group have responsibility for asylum seeker housing in Glasgow.

2 Ministerial decision 186/2017 pursuant to Article 9Θ(1)(b) of the Refugee Law

3 Inspected upon visiting the Kofinou Centre on 17 May 2018

4 As the findings from the Prospects for International Migration Governance (MIGPROSP) project has shown, in national level migration policy ‘not only is change seen as difficult to deliver, but change itself is viewed as problematic because of the possibility of unforeseen consequences in an unstable and highly politicised policy field’ (Geddes 2016).
